# Centroid based clustering of high throughput sequencing reads based on *n*-mer counts

**DOI:** 10.1186/1471-2105-14-268

**Published:** 2013-09-08

**Authors:** Alexander Solovyov, W Ian Lipkin

**Affiliations:** 1 Center for Infection and Immunity, Columbia University, New York, NY, 10032, USA

## Abstract

**Background:**

Many problems in computational biology require alignment-free sequence comparisons. One of the common tasks involving sequence comparison is sequence clustering. Here we apply methods of alignment-free comparison (in particular, comparison using sequence composition) to the challenge of sequence clustering.

**Results:**

We study several centroid based algorithms for clustering sequences based on word counts. Study of their performance shows that using *k*-means algorithm with or without the data whitening is efficient from the computational point of view. A higher clustering accuracy can be achieved using the soft expectation maximization method, whereby each sequence is attributed to each cluster with a specific probability. We implement an open source tool for alignment-free clustering. It is publicly available from github: https://github.com/luscinius/afcluster.

**Conclusions:**

We show the utility of alignment-free sequence clustering for high throughput sequencing analysis despite its limitations. In particular, it allows one to perform assembly with reduced resources and a minimal loss of quality. The major factor affecting performance of alignment-free read clustering is the length of the read.

## Background

The most common technique for establishing a homology between sequences is sequence alignment (e. g., [1]). Numerous algorithms have been developed for aligning sequences. These include exhaustive dynamic programming algorithms as well as faster heuristic algorithms (e. g., BLAST [2]). In these algorithms each alignment is evaluated using some score matrix, wherein the score matrix depends on the expected similarity between the aligned sequences. However, in some cases one needs to compare related sequences that are divergent, or related sequences that are not at all homologous. One example of biologically related sequences that do not share a common ancestor is where two genes with different evolutionary history are found on the same genome. Another example is related to sequencing wherein non-overlapping reads originate from the same gene (“contig”). These cases do not allow for a direct sequence alignment. They may, however, be identified as related using alignment-free sequence comparisons.

Alignment-free methods are less accurate than direct sequence alignments. Thus, they are only used as a last resort when direct alignment is either impossible or computationally complex. A common method for alignment-free sequence comparison is comparison via the word (*n*-mer) counts [3-5]. In this approach a nucleotide sequence of arbitrary length *L* is represented by the counts of the 4^*n*^ different *n*-mers. It is no surprise that such comparisons are less accurate than the sequence alignment as replacing the sequence with the vector of the word counts results in a loss of information.

High throughput sequencing data analysis is a relatively novel area of computational biology where application of alignment-free sequence comparison is especially desirable. Indeed, high throughput sequencing runs generate a vast amount of relatively short (typically 30-500 bp) reads. These factors make direct comparison with the reference complex if not impossible. One can think of different applications of alignment-free methods to sequencing data. In particular, an assembly free comparison of genomes using reads generated from them is possible [6]. In the present work we focus on a different application: clustering of reads coming from different genes and possibly different species based on *n*-mer counts. One case when such a clustering is desirable is a sample of large size, so that the large number of reads makes direct assembly computationally prohibitive. In this case clustering reads rather than randomly splitting the sample is desirable.

In the present study we focus on small values of *n*, such that *L* ≫ 4^*n*^. In other words, we assume that the compared sequences are sufficiently long to avoid the situation where *L* ≤ 4^*n*^ and almost all counts are either zero or one. In particular, for 30 bp reads the appropriate value of *n* is 1, possibly 2; for 400 bp reads the appropriate values of *n* are 1, 2 or 3.

We implement a word count based clustering algorithm for short nucleotide sequences (“reads”)^1^. In this approach each sequence is represented by the vector of the *n*-mer counts (or *n*-mer frequencies). We focus on a centroid based clustering algorithm because of its linear space and time complexity. In the framework of centroid based clustering each centroid is characterized by its *n*-mer frequencies. In particular, we study expectation maximization (EM) algorithm, which is a generalization of the *k*-means algorithm. Each individual sequence is attributed to a centroid based on the likelihood of obtaining the observed word counts within a given model. In this context the Kullback-Leibler (KL) divergence naturally arises as the log likelihood function. We study centroid based algorithms involving other distances as well. We evaluate performance of different algorithms using simulated reads data. In the end we apply our clustering methods to a real sequencing run.

## Results and discussion

### Comparison of centroid based algorithms

We study several centroid based clustering algorithms in the context of alignment-free sequence comparison. From the point of view of these algorithms each sequence is represented by the vector of the word (*n*-mer) counts. We restrict ourselves to the case of the relatively short sequences, having length typical to sequencer reads. We implement expectation maximization algorithm using Kullback-Leibler divergence as the log likelihood function. We also consider centroid based clustering algorithms using *L*_2_ distance (regular distance in Euclidean space) and *d*_2_ distance [6]. In addition we consider *k*-means algorithm. *k*-means algorithm is the *L*_2_ algorithm with preliminary whitening of data. All these algorithms have an established convergence property. We implement centroid based clustering algorithms using some other distance functions: symmetrized KL divergence, $d_{2}^{*}$[6] and *χ*^2^ statistic (see Methods section for a detailed description). The latter algorithms do not possess a known convergence property.

We compared the performance of these algorithms using 50 randomly chosen subsets of human reference mRNA sequences. Each subset consisted of 1000 sequences. For each of the 50 subsets we generated several sets of simulated reads, different sets containing simulated reads of different length. For each dataset we performed clustering using different methods. For *k*-means clustering we used implementation available in *scipy* package. We evaluated classification performance (recall) for a sequence as the maximal fraction of reads from this sequence which fall into the same cluster. We compared the distribution of the recall rates obtained for each sequence in each of the datasets. The results are presented in Tables 1, 2, 3, and 4. In this simulation the EM algorithm showed a higher performance for the word size *n* = 1 with 4 or 5 clusters, *n* = 2 with 2 clusters and *n* = 3 with 2 clusters. The *L*_2_ algorithm showed a higher performance for *n* = 2 with 4 or 5 clusters and for *n* = 3 with 4 or 5 clusters. Note that the *L*_2_ and *d*_2_ algorithms operate with the word frequencies normalized for each read individually, while the *k*-means and the EM methods use the normalization related to several reads (cf. Equation (5)). Methods from the first group (*L*_2_ and *d*_2_) generally exhibit a better performance for a larger number of clusters.

**Table 1:** **Recall rates for simulated human reads of different length,** ***n*** **= 1**

	**EM**		***k*-means**		***L***_***2***_		***d***_***2***_	
**Read length**	**Recall**	**std. dev.**	**Recall**	**std. dev.**	**Recall**	**std. dev.**	**Recall*t***	**std. dev.**
2 clusters								
30	0.740	0.133	0.740	0.134	0.740	0.133	0.738	0.134
50	0.763	0.142	0.763	0.142	0.763	0.141	0.763	0.142
75	0.781	0.146	0.781	0.146	0.781	0.146	0.781	0.146
100	0.794	0.148	0.794	0.149	0.794	0.148	0.794	0.148
150	0.812	0.152	0.811	0.153	0.812	0.152	0.812	0.152
200	0.827	0.153	0.826	0.154	0.827	0.153	0.827	0.153
250	0.839	0.154	0.839	0.154	0.840	0.153	0.840	0.153
300	0.850	0.153	0.850	0.154	0.850	0.153	0.850	0.153
400	0.868	0.152	0.868	0.152	0.868	0.152	0.868	0.152
3 clusters								
30	0.581	0.118	0.582	0.131	0.580	0.119	0.575	0.120
50	0.608	0.130	0.609	0.136	0.606	0.129	0.606	0.136
75	0.631	0.138	0.632	0.143	0.630	0.141	0.631	0.144
100	0.648	0.144	0.650	0.149	0.648	0.145	0.647	0.148
150	0.676	0.154	0.677	0.157	0.675	0.155	0.675	0.157
200	0.697	0.162	0.697	0.164	0.697	0.162	0.697	0.163
250	0.715	0.168	0.715	0.170	0.715	0.168	0.715	0.169
300	0.731	0.171	0.731	0.173	0.731	0.172	0.732	0.173
400	0.758	0.177	0.757	0.178	0.757	0.177	0.758	0.178
4 clusters								
30	0.489	0.104	0.484	0.116	0.488	0.105	0.478	0.107
50	0.519	0.114	0.512	0.118	0.513	0.117	0.509	0.118
75	0.542	0.126	0.537	0.130	0.539	0.126	0.534	0.129
100	0.562	0.132	0.556	0.136	0.558	0.133	0.554	0.135
150	0.590	0.145	0.587	0.150	0.587	0.145	0.585	0.147
200	0.612	0.155	0.611	0.159	0.612	0.155	0.609	0.156
250	0.633	0.163	0.633	0.167	0.631	0.163	0.630	0.165
300	0.652	0.170	0.650	0.174	0.650	0.171	0.649	0.171
400	0.683	0.180	0.682	0.184	0.682	0.180	0.681	0.181
5 clusters								
30	0.436	0.099	0.431	0.106	0.431	0.100	0.426	0.104
50	0.459	0.108	0.450	0.115	0.455	0.108	0.446	0.109
75	0.480	0.117	0.470	0.122	0.475	0.118	0.470	0.121
100	0.499	0.126	0.493	0.130	0.495	0.126	0.488	0.128
150	0.530	0.139	0.524	0.142	0.528	0.139	0.522	0.141
200	0.556	0.151	0.550	0.154	0.552	0.150	0.548	0.153
250	0.577	0.160	0.572	0.163	0.572	0.160	0.570	0.160
300	0.596	0.168	0.592	0.171	0.594	0.168	0.590	0.169
400	0.630	0.181	0.626	0.185	0.629	0.181	0.626	0.181

Mean recall rates and standard deviation for various read lengths and numbers of clusters. For every read length clustering was performed on 50 simulated read sets, each set originating from 1000 randomly chosen human RNA reference sequences and having 100000 reads. Word length is *n* = 1.

**Table 2:** **Recall rates for simulated human reads of different length,** ***n*** **= 2**

	**EM**		***k*-means**		***L***_***2***_		***d***_***2***_	
**Read length**	**Recall**	**std. dev.**	**Recall**	**std. dev.**	**Recall**	**std. dev.**	**Recall*t***	**std. dev.**
2 clusters								
30	0.737	0.133	0.735	0.136	0.735	0.137	0.735	0.136
50	0.762	0.141	0.760	0.143	0.760	0.143	0.759	0.142
75	0.781	0.145	0.778	0.147	0.778	0.147	0.778	0.147
100	0.794	0.148	0.791	0.150	0.791	0.149	0.791	0.149
150	0.812	0.152	0.810	0.153	0.810	0.153	0.810	0.153
200	0.827	0.153	0.825	0.155	0.825	0.154	0.825	0.154
250	0.839	0.153	0.837	0.155	0.837	0.155	0.837	0.155
300	0.850	0.153	0.848	0.155	0.848	0.155	0.848	0.155
400	0.867	0.152	0.866	0.154	0.867	0.154	0.867	0.154
3 clusters								
30	0.573	0.110	0.573	0.108	0.572	0.106	0.567	0.108
50	0.604	0.124	0.603	0.126	0.602	0.122	0.600	0.124
75	0.629	0.135	0.629	0.138	0.627	0.134	0.626	0.136
100	0.647	0.142	0.647	0.146	0.645	0.142	0.644	0.144
150	0.675	0.153	0.675	0.156	0.673	0.153	0.673	0.155
200	0.696	0.160	0.696	0.164	0.695	0.161	0.694	0.162
250	0.714	0.166	0.714	0.170	0.713	0.167	0.713	0.168
300	0.730	0.171	0.730	0.173	0.730	0.171	0.729	0.172
400	0.756	0.177	0.757	0.179	0.756	0.177	0.756	0.178
4 clusters								
30	0.492	0.096	0.492	0.097	0.497	0.096	0.473	0.112
50	0.523	0.109	0.526	0.110	0.530	0.110	0.521	0.110
75	0.549	0.121	0.550	0.122	0.557	0.123	0.550	0.122
100	0.567	0.129	0.567	0.131	0.576	0.131	0.570	0.131
150	0.596	0.143	0.595	0.147	0.603	0.144	0.599	0.144
200	0.618	0.153	0.616	0.157	0.624	0.154	0.620	0.154
250	0.638	0.161	0.637	0.166	0.643	0.162	0.640	0.162
300	0.655	0.168	0.654	0.173	0.658	0.168	0.656	0.168
400	0.684	0.179	0.685	0.184	0.688	0.179	0.686	0.179
5 clusters								
30	0.418	0.108	0.411	0.102	0.409	0.103	0.395	0.109
50	0.456	0.122	0.465	0.114	0.474	0.109	0.455	0.123
75	0.492	0.119	0.498	0.123	0.501	0.122	0.493	0.121
100	0.513	0.128	0.518	0.133	0.522	0.132	0.516	0.131
150	0.546	0.142	0.550	0.146	0.555	0.146	0.550	0.145
200	0.569	0.153	0.572	0.157	0.578	0.156	0.574	0.156
250	0.589	0.162	0.592	0.165	0.600	0.165	0.595	0.165
300	0.607	0.169	0.609	0.172	0.618	0.172	0.614	0.172
400	0.638	0.181	0.638	0.184	0.648	0.183	0.645	0.183

Mean recall rates and standard deviation for various read lengths and numbers of clusters. For every read length clustering was performed on 50 simulated read sets, each set originating from 1000 randomly chosen human RNA reference sequences and having 100000 reads. Word length is *n* = 2.

**Table 3:** **Recall rates for simulated human reads of different length,** ***n*** **= 3**

	**EM**		***k*-means**		***L***_***2***_		***d***_***2***_	
**Read length**	**Recall**	**std. dev.**	**Recall**	**std. dev.**	**Recall**	**std. dev.**	**Recall**	**std. dev.**
2 clusters								
30	0.734	0.134	0.733	0.139	0.734	0.141	0.734	0.139
50	0.761	0.141	0.757	0.144	0.758	0.145	0.757	0.144
75	0.780	0.145	0.775	0.148	0.775	0.148	0.775	0.148
100	0.793	0.148	0.789	0.150	0.789	0.150	0.789	0.150
150	0.811	0.152	0.808	0.154	0.808	0.154	0.808	0.153
200	0.827	0.153	0.822	0.155	0.823	0.155	0.823	0.155
250	0.839	0.153	0.835	0.156	0.835	0.155	0.836	0.155
300	0.850	0.153	0.846	0.155	0.846	0.155	0.847	0.155
400	0.867	0.152	0.865	0.155	0.865	0.154	0.865	0.154
3 clusters								
30	0.569	0.109	0.582	0.111	0.587	0.113	0.577	0.113
50	0.601	0.124	0.608	0.128	0.608	0.127	0.601	0.127
75	0.628	0.135	0.632	0.141	0.629	0.138	0.625	0.138
100	0.646	0.142	0.649	0.148	0.646	0.144	0.643	0.145
150	0.674	0.153	0.675	0.158	0.673	0.155	0.671	0.156
200	0.696	0.160	0.696	0.166	0.693	0.162	0.692	0.163
250	0.714	0.166	0.714	0.171	0.712	0.168	0.711	0.169
300	0.730	0.171	0.731	0.175	0.729	0.172	0.728	0.173
400	0.756	0.177	0.757	0.180	0.755	0.178	0.755	0.179
4 clusters								
30	0.465	0.117	0.495	0.097	0.518	0.098	0.497	0.098
50	0.529	0.112	0.543	0.114	0.553	0.116	0.541	0.116
75	0.556	0.124	0.569	0.127	0.580	0.130	0.570	0.130
100	0.575	0.131	0.584	0.135	0.599	0.140	0.591	0.139
150	0.602	0.145	0.607	0.148	0.625	0.151	0.619	0.151
200	0.623	0.153	0.626	0.158	0.644	0.159	0.640	0.159
250	0.642	0.161	0.642	0.166	0.659	0.164	0.657	0.165
300	0.658	0.168	0.657	0.173	0.672	0.169	0.670	0.170
400	0.687	0.178	0.687	0.183	0.695	0.179	0.693	0.179
5 clusters								
30	0.411	0.105	0.410	0.094	0.416	0.092	0.405	0.093
50	0.454	0.126	0.481	0.120	0.509	0.125	0.493	0.127
75	0.492	0.121	0.506	0.123	0.516	0.127	0.504	0.127
100	0.516	0.130	0.526	0.132	0.528	0.132	0.520	0.132
150	0.550	0.144	0.557	0.147	0.560	0.146	0.553	0.146
200	0.573	0.155	0.581	0.158	0.584	0.157	0.578	0.158
250	0.595	0.164	0.603	0.167	0.605	0.166	0.601	0.167
300	0.613	0.171	0.622	0.174	0.625	0.173	0.620	0.173
400	0.644	0.182	0.652	0.185	0.656	0.184	0.653	0.185

Mean recall rates and standard deviation for various read lengths and numbers of clusters. For every read length clustering was performed on 50 simulated read sets, each set originating from 1000 randomly chosen human RNA reference sequences and having 100000 reads. Word length is *n* = 3.

**Table 4:** **Recall rates for simulated human reads of different length, various distance functions,** ***n*** **= 2**

	**EM**		***k*-means**		$d_{2}^{*}$		***χ***^***2***^		**Symmetrized KL**	
**Read length**	**Recall**	**std. dev.**	**Recall**	**std. dev.**	**Recall**	**std. dev.**	**Recall**	**std. dev.**	**Recall**	**std. dev.**
2 clusters										
30	0.737	0.133	0.735	0.136	0.610	0.083	0.737	0.140	0.736	0.134
50	0.762	0.141	0.760	0.143	0.649	0.105	0.760	0.144	0.762	0.141
75	0.781	0.145	0.778	0.147	0.677	0.122	0.778	0.148	0.781	0.145
100	0.794	0.148	0.791	0.150	0.719	0.131	0.791	0.150	0.794	0.148
150	0.812	0.152	0.810	0.153	0.803	0.147	0.810	0.154	0.812	0.152
200	0.827	0.153	0.825	0.155	0.824	0.151	0.824	0.155	0.826	0.153
250	0.839	0.153	0.837	0.155	0.838	0.151	0.837	0.156	0.839	0.153
300	0.850	0.153	0.848	0.155	0.850	0.152	0.847	0.156	0.850	0.153
400	0.867	0.152	0.866	0.154	0.869	0.152	0.866	0.154	0.867	0.152
3 clusters										
30	0.573	0.110	0.573	0.108	0.447	0.076	0.715	0.131	0.572	0.111
50	0.604	0.124	0.603	0.126	0.474	0.090	0.674	0.134	0.603	0.125
75	0.629	0.135	0.629	0.138	0.626	0.139	0.664	0.144	0.629	0.136
100	0.647	0.142	0.647	0.146	0.671	0.148	0.668	0.150	0.647	0.143
150	0.675	0.153	0.675	0.156	0.724	0.157	0.687	0.159	0.675	0.153
200	0.696	0.160	0.696	0.164	0.692	0.161	0.706	0.167	0.696	0.160
250	0.714	0.166	0.714	0.170	0.714	0.166	0.723	0.172	0.714	0.166
300	0.730	0.171	0.730	0.173	0.730	0.170	0.738	0.176	0.730	0.170
400	0.756	0.177	0.757	0.179	0.757	0.176	0.762	0.180	0.756	0.176

Mean recall rates and standard deviation for various read lengths and 2 or 3 clusters. For every read length clustering was performed on 50 simulated read sets, each set originating from 1000 randomly chosen human RNA reference sequences and having 100000 reads. Clustering was performed using all distance functions considered in the paper, including those which do not guarantee convergence. Results for *L*_2_ and *d*_2_ distance are not shown. Word length is *n* = 2.

Even though these differences can be considered statistically significant, their magnitude is rather small for practical purposes. Based on this fact we recommend using the *L*_2_ or *k*-means algorithm for computational efficiency. Indeed, the EM algorithm involves the computationally expensive evaluation of the natural logarithms; while the *L*_2_ and *k*-means algorithms only involve arithmetic operations. This can make the run time of the EM algorithm exceed that of the *L*_2_ and *k*-means algorithms by more than a factor of 10.

We evaluated the performance of the algorithms involving the symmetrized KL divergence, $d_{2}^{*}$ and the *χ*^2^ distance on the same datasets for the word length *n* = 2. The $d_{2}^{*}$ algorithm failed to converge in 21 out of 900 runs. The results are shown in Table 4. None of the three mentioned algorithms exhibits a performance better than that of the EM or *k*-means algorithm. Taking into account the fact that the convergence of these algorithms is not established (and the numerical experiment in fact proves the lack of guaranteed convergence for the $d_{2}^{*}$ algorithm), our data exhibit no benefits of using these methods.

Tables 1, 2, 3 and 4 show that the recall rate increases with the increasing read length, number of reads being constant. This conforms to our intuition that with the increasing reads length the word counts increase, resulting in a smaller effect of statistical fluctuations. Tables 1, 2 and 3 show that the recall rate has almost no dependence on the word size *n* for *n* = 1,2,3.

We performed a set of simulations with different number of reads generated from the same source sequences in order to study the dependence of the recall rate on the number of reads. The results are shown in Table 5. For smaller number of reads the recall rate is lower. It is gradually increasing and stabilizing as the number of reads is increasing. Our explanation for this fact is that for a small number of reads some of the source sequences have only a few reads, and the recall rate is significantly influenced by the pseudocounts.

**Table 5:** **Recall rates for simulated human reads, different number of reads,** ***n*** **= 2**

	**EM**		***L***_***2***_		***d***_***2***_	
**Number of reads**	**Recall**	**std. dev.**	**Recall**	**std. dev.**	**Recall**	**std. dev.**
2 clusters						
5000	0.783	0.160	0.793	0.166	0.790	0.165
10000	0.787	0.151	0.793	0.156	0.793	0.156
20000	0.798	0.146	0.801	0.151	0.801	0.150
30000	0.805	0.146	0.806	0.150	0.806	0.150
50000	0.812	0.147	0.812	0.150	0.812	0.149
75000	0.815	0.148	0.815	0.151	0.815	0.150
100000	0.818	0.149	0.816	0.151	0.816	0.151
150000	0.820	0.150	0.819	0.152	0.819	0.151
200000	0.821	0.150	0.819	0.152	0.819	0.152
400000	0.823	0.151	0.821	0.153	0.821	0.152
3 clusters						
5000	0.657	0.181	0.660	0.184	0.656	0.181
10000	0.653	0.162	0.655	0.164	0.653	0.163
20000	0.661	0.151	0.661	0.153	0.659	0.152
30000	0.667	0.149	0.667	0.150	0.665	0.150
50000	0.674	0.150	0.674	0.151	0.673	0.152
75000	0.679	0.152	0.678	0.153	0.677	0.153
100000	0.682	0.153	0.681	0.154	0.680	0.155
150000	0.685	0.154	0.684	0.155	0.683	0.156
200000	0.686	0.155	0.685	0.156	0.685	0.157
400000	0.689	0.156	0.688	0.157	0.687	0.158
4 clusters						
5000	0.577	0.183	0.587	0.189	0.581	0.188
10000	0.569	0.159	0.577	0.163	0.573	0.162
20000	0.576	0.144	0.583	0.146	0.580	0.145
30000	0.583	0.141	0.590	0.143	0.586	0.142
50000	0.591	0.140	0.598	0.142	0.595	0.142
75000	0.597	0.142	0.603	0.144	0.599	0.143
100000	0.600	0.143	0.606	0.145	0.603	0.145
150000	0.604	0.145	0.610	0.146	0.607	0.146
200000	0.605	0.145	0.612	0.147	0.608	0.147
400000	0.608	0.147	0.615	0.148	0.611	0.148
5 clusters						
5000	0.520	0.181	0.534	0.187	0.527	0.184
10000	0.514	0.156	0.527	0.162	0.520	0.158
20000	0.521	0.140	0.532	0.145	0.527	0.144
30000	0.529	0.138	0.540	0.142	0.535	0.141
50000	0.539	0.139	0.549	0.143	0.544	0.142
75000	0.545	0.140	0.555	0.144	0.550	0.144
100000	0.548	0.142	0.558	0.146	0.553	0.145
150000	0.552	0.144	0.562	0.148	0.557	0.147
200000	0.554	0.145	0.564	0.149	0.560	0.148
400000	0.558	0.146	0.568	0.150	0.563	0.150

Mean recall rates and standard deviation for different number of reads. For each of the 50 randomly chosen subsets of human reference RNA sequences we simulated reads, choosing the specified number of reads. Clustering was performed using the EM, *L*_2_ and *d*_2_ algorithms. Word length is *n* = 2. Read length is 200bp. When computing the recall rate for each contig we use pseudocounts, artificially increasing the count of reads in each cluster by one.

We performed a series of simulations for different sequencing error rates. The results are shown in Tables 6, 7 and 8. As expected, the recall rate decreases monotonically when the sequencing error rate increases for all clustering methods.

**Table 6:** **Recall rates for simulated human reads, *n*** **= 1, different error rates**

	**EM**		***k*-means**		***L***_***2***_		***d***_***2***_	
**Error rate**	**Recall**	**std. dev.**	**Recall**	**std. dev.**	**Recall**	**std. dev.**	**Recall**	**std. dev.**
2 clusters								
0	0.827	0.153	0.826	0.154	0.827	0.153	0.827	0.153
0.001	0.827	0.153	0.827	0.153	0.828	0.153	0.828	0.153
0.005	0.827	0.153	0.826	0.153	0.827	0.153	0.827	0.153
0.01	0.826	0.153	0.826	0.153	0.827	0.153	0.827	0.153
0.02	0.826	0.152	0.825	0.153	0.826	0.152	0.826	0.152
0.03	0.825	0.153	0.825	0.153	0.825	0.152	0.825	0.152
0.04	0.824	0.152	0.824	0.153	0.825	0.152	0.825	0.152
0.05	0.824	0.153	0.824	0.153	0.824	0.152	0.824	0.152
3 clusters								
0	0.697	0.162	0.697	0.164	0.697	0.162	0.697	0.163
0.001	0.697	0.162	0.697	0.163	0.697	0.162	0.697	0.163
0.005	0.696	0.160	0.696	0.163	0.696	0.162	0.696	0.162
0.01	0.695	0.160	0.696	0.163	0.695	0.161	0.695	0.162
0.02	0.694	0.160	0.694	0.162	0.694	0.160	0.694	0.162
0.03	0.693	0.159	0.693	0.161	0.693	0.160	0.693	0.161
0.04	0.691	0.159	0.692	0.161	0.691	0.159	0.691	0.161
0.05	0.691	0.157	0.691	0.160	0.691	0.158	0.690	0.160
4 clusters								
0	0.612	0.155	0.611	0.159	0.612	0.155	0.609	0.156
0.001	0.613	0.153	0.611	0.159	0.611	0.154	0.609	0.156
0.005	0.613	0.154	0.610	0.158	0.611	0.155	0.609	0.156
0.01	0.611	0.153	0.610	0.157	0.609	0.153	0.608	0.155
0.02	0.610	0.152	0.607	0.156	0.608	0.152	0.606	0.154
0.03	0.608	0.150	0.606	0.155	0.607	0.151	0.604	0.152
0.04	0.606	0.150	0.604	0.156	0.605	0.150	0.603	0.152
0.05	0.605	0.149	0.603	0.154	0.604	0.150	0.601	0.151
5 clusters								
0	0.556	0.151	0.550	0.154	0.552	0.150	0.548	0.153
0.001	0.556	0.151	0.549	0.154	0.551	0.151	0.547	0.151
0.005	0.554	0.150	0.549	0.153	0.551	0.149	0.549	0.151
0.01	0.554	0.148	0.547	0.152	0.552	0.148	0.547	0.149
0.02	0.553	0.148	0.544	0.150	0.550	0.147	0.546	0.149
0.03	0.552	0.146	0.544	0.149	0.548	0.146	0.544	0.146
0.04	0.549	0.145	0.542	0.149	0.546	0.144	0.543	0.146
0.05	0.548	0.143	0.540	0.149	0.545	0.144	0.541	0.145

Mean recall rates and standard deviation for various error rates and numbers of clusters. For every value of the error rate clustering was performed on 50 simulated read sets, each set originating from 1000 randomly chosen human RNA reference sequences and having 100000 reads. Word length is *n* = 1. Read length is 200bp.

**Table 7:** **Recall rates for simulated human reads, *n*** **= 2, different error rates**

	**EM**		***k*-means**		***L***_***2***_		***d***_***2***_	
**Error rate**	**Recall**	**std. dev.**	**Recall**	**std. dev.**	**Recall**	**std. dev.**	**Recall**	**std. dev.**
2 clusters								
0	0.827	0.153	0.825	0.155	0.825	0.154	0.825	0.154
0.001	0.827	0.152	0.825	0.155	0.825	0.154	0.825	0.154
0.005	0.826	0.153	0.824	0.154	0.824	0.154	0.825	0.154
0.01	0.826	0.152	0.824	0.154	0.824	0.154	0.824	0.154
0.02	0.825	0.152	0.823	0.154	0.824	0.154	0.824	0.154
0.03	0.825	0.152	0.823	0.154	0.823	0.154	0.823	0.154
0.04	0.824	0.152	0.822	0.154	0.822	0.154	0.822	0.154
0.05	0.823	0.152	0.822	0.154	0.822	0.154	0.822	0.154
3 clusters								
0	0.696	0.160	0.696	0.164	0.695	0.161	0.694	0.162
0.001	0.696	0.160	0.696	0.163	0.695	0.161	0.694	0.162
0.005	0.695	0.160	0.695	0.163	0.694	0.160	0.694	0.162
0.01	0.695	0.159	0.695	0.164	0.693	0.160	0.693	0.161
0.02	0.694	0.159	0.694	0.162	0.693	0.160	0.692	0.161
0.03	0.693	0.158	0.692	0.161	0.691	0.159	0.691	0.160
0.04	0.691	0.158	0.691	0.161	0.690	0.159	0.689	0.160
0.05	0.690	0.157	0.690	0.160	0.689	0.157	0.689	0.159
4 clusters								
0	0.618	0.153	0.616	0.157	0.624	0.154	0.620	0.154
0.001	0.618	0.152	0.617	0.158	0.624	0.153	0.621	0.153
0.005	0.617	0.152	0.616	0.157	0.623	0.153	0.620	0.153
0.01	0.616	0.152	0.615	0.156	0.622	0.152	0.618	0.152
0.02	0.614	0.151	0.613	0.155	0.620	0.151	0.616	0.151
0.03	0.612	0.149	0.612	0.154	0.618	0.150	0.614	0.150
0.04	0.611	0.149	0.610	0.153	0.616	0.149	0.612	0.150
0.05	0.609	0.148	0.609	0.152	0.614	0.149	0.610	0.149
5 clusters								
0	0.569	0.153	0.572	0.157	0.578	0.156	0.574	0.156
0.001	0.569	0.153	0.572	0.157	0.579	0.156	0.574	0.156
0.005	0.568	0.152	0.572	0.156	0.578	0.155	0.573	0.156
0.01	0.567	0.151	0.571	0.155	0.577	0.155	0.572	0.155
0.02	0.565	0.150	0.568	0.154	0.575	0.154	0.570	0.154
0.03	0.563	0.149	0.566	0.152	0.573	0.153	0.569	0.152
0.04	0.560	0.147	0.563	0.152	0.570	0.151	0.565	0.151
0.05	0.559	0.147	0.561	0.150	0.569	0.150	0.564	0.150

Mean recall rates and standard deviation for various error rates and numbers of clusters. For every value of the error rate clustering was performed on 50 simulated read sets, each set originating from 1000 randomly chosen human RNA reference sequences and having 100000 reads. Word length is *n* = 2. Read length is 200bp.

**Table 8:** **Recall rates for simulated human reads, *n*** **= 3, different error rates**

	**EM**		***k*-means**		***L***_***2***_		***d***_***2***_	
**Error rate**	**Recall**	**std. dev.**	**Recall**	**std. dev.**	**Recall**	**std. dev.**	**Recall**	**std. dev.**
2 clusters								
0	0.827	0.153	0.822	0.155	0.823	0.155	0.823	0.155
0.001	0.827	0.152	0.823	0.155	0.823	0.155	0.823	0.155
0.005	0.826	0.153	0.822	0.155	0.823	0.155	0.823	0.155
0.01	0.826	0.152	0.822	0.155	0.822	0.154	0.822	0.154
0.02	0.825	0.152	0.821	0.155	0.822	0.155	0.822	0.154
0.03	0.825	0.152	0.821	0.155	0.821	0.155	0.821	0.154
0.04	0.824	0.152	0.820	0.155	0.820	0.154	0.820	0.154
0.05	0.823	0.152	0.819	0.155	0.820	0.155	0.820	0.154
3 clusters								
0	0.696	0.160	0.696	0.166	0.693	0.162	0.692	0.163
0.001	0.696	0.160	0.697	0.166	0.694	0.162	0.692	0.163
0.005	0.695	0.160	0.695	0.164	0.693	0.161	0.692	0.162
0.01	0.694	0.159	0.695	0.164	0.692	0.161	0.691	0.162
0.02	0.693	0.159	0.693	0.164	0.691	0.161	0.690	0.162
0.03	0.692	0.158	0.693	0.164	0.690	0.160	0.689	0.161
0.04	0.691	0.158	0.691	0.163	0.688	0.160	0.687	0.161
0.05	0.690	0.157	0.690	0.162	0.687	0.158	0.686	0.159
4 clusters								
0	0.623	0.153	0.626	0.158	0.644	0.159	0.640	0.159
0.001	0.624	0.153	0.625	0.158	0.644	0.158	0.640	0.158
0.005	0.622	0.153	0.625	0.157	0.644	0.158	0.639	0.158
0.01	0.621	0.152	0.623	0.156	0.642	0.157	0.637	0.157
0.02	0.619	0.151	0.622	0.155	0.638	0.156	0.635	0.156
0.03	0.617	0.149	0.618	0.153	0.636	0.154	0.632	0.154
0.04	0.615	0.149	0.615	0.152	0.632	0.153	0.628	0.153
0.05	0.613	0.148	0.613	0.152	0.629	0.152	0.625	0.151
5 clusters								
0	0.573	0.155	0.581	0.158	0.584	0.157	0.578	0.158
0.001	0.574	0.155	0.582	0.158	0.584	0.157	0.578	0.158
0.005	0.573	0.154	0.581	0.156	0.583	0.156	0.578	0.157
0.01	0.572	0.153	0.580	0.156	0.582	0.155	0.577	0.156
0.02	0.570	0.152	0.578	0.156	0.580	0.155	0.575	0.155
0.03	0.568	0.150	0.576	0.154	0.578	0.153	0.573	0.154
0.04	0.565	0.149	0.572	0.151	0.575	0.152	0.569	0.152
0.05	0.563	0.148	0.571	0.151	0.574	0.151	0.568	0.151

Mean recall rates and standard deviation for various error rates and numbers of clusters. For every value of the error rate clustering was performed on 50 simulated read sets, each set originating from 1000 randomly chosen human RNA reference sequences and having 100000 reads. Word length is *n* = 3. Read length is 200bp.

### Soft EM clustering

**Figure 1:**
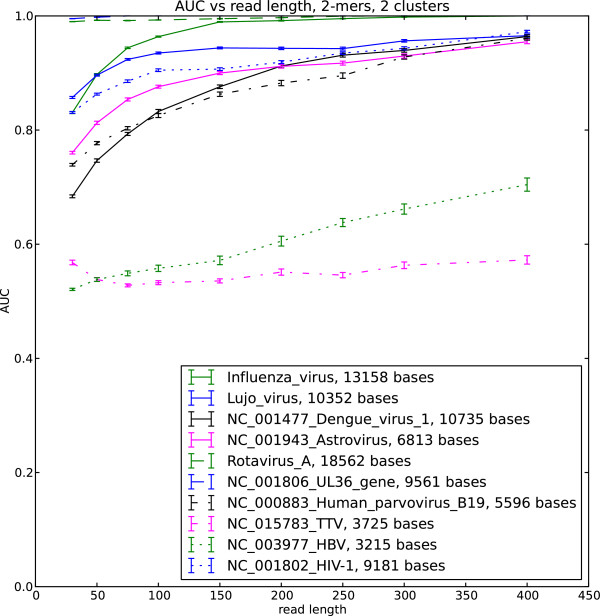
**AUC for selected viral sequences, 2 clusters.** Viral reads clustered with background human reads, AUC is calculated for each virus.

**Figure 2:**
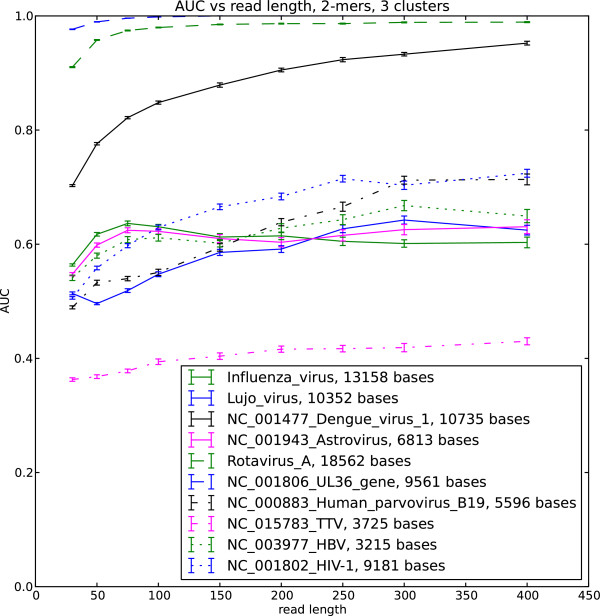
**AUC for selected viral sequences, 3 clusters.** Viral reads clustered with background human reads, AUC is calculated for each virus.

**Figure 3:**
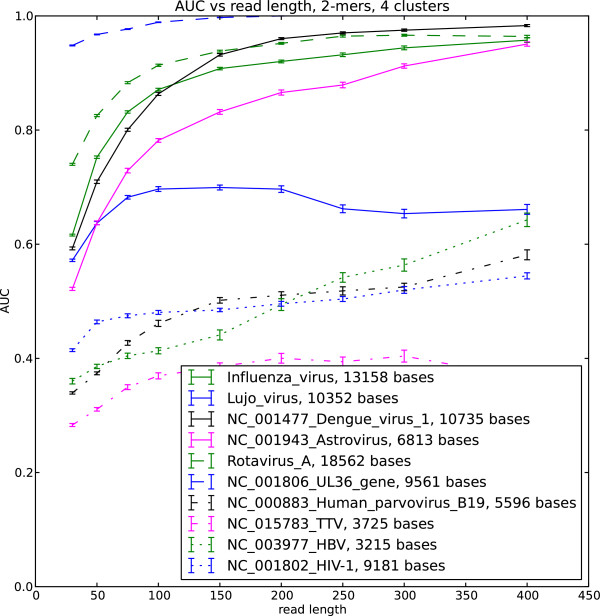
**AUC for selected viral sequences, 4 clusters.** Viral reads clustered with background human reads, AUC is calculated for each virus.

**Figure 4:**
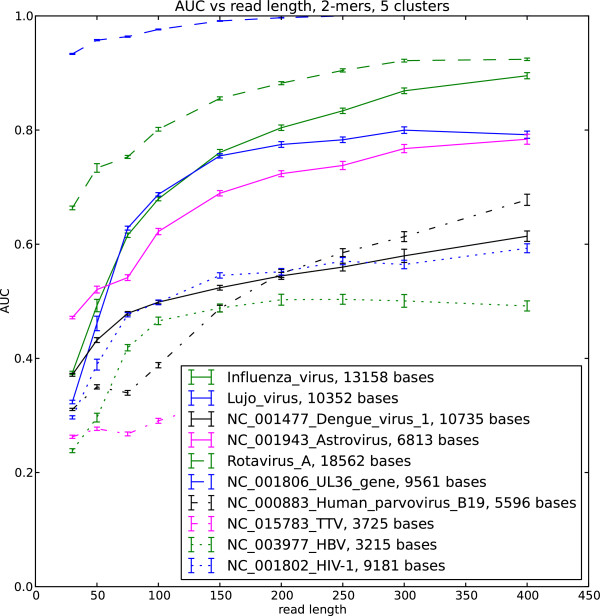
**AUC for selected viral sequences, 5 clusters.** Viral reads clustered with background human reads, AUC is calculated for each virus.

We implemented the soft EM clustering algorithm using the KL divergence as the log likelihood function. We performed soft EM clustering of simulated viral reads in the human background using single stranded and double stranded DNA and RNA viruses as well as retroviruses. We generated 50 datasets for each read length and built the ROC curve for each dataset. The area under the curve (AUC) averaged over the 50 datasets is shown in Figure 1, 2, 3 and 4

**Figure 5:**
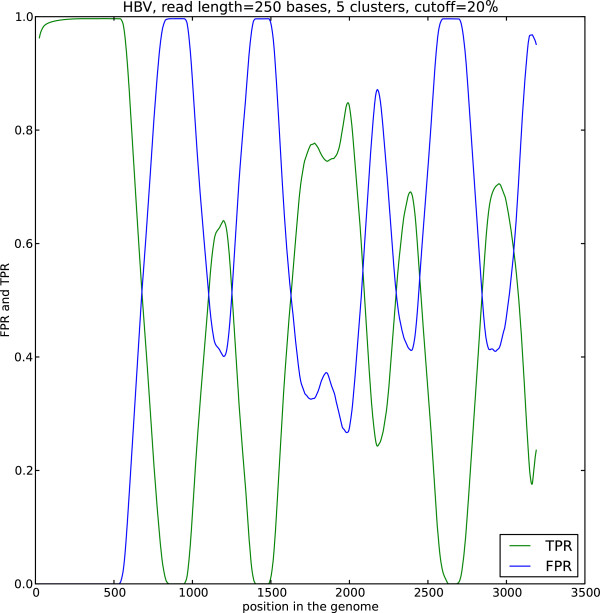
**Assignment of reads depending on position.** The fraction of reads assigned to the dominant (TPR) and other than the dominant (FPR) cluster as a function of the position in the genome of the Hepatitis B virus. The data are smoothed by averaging over the window of length 50. Different regions of the genome cluster differently, forming consistent patterns as a consequence of the changing nucleotide composition across the genome.

The AUC and its dependence on the read length is determined by the interplay of the different factors. These include the choice of the likelihood function in the EM algorithm, uniformity of the sequence composition of the studied viral sequences as well as the choice of the background reads. Our results indicate that double stranded viruses as well as single stranded RNA viruses generally show higher AUC than single stranded DNA and retroviruses. Note that the lower AUC is a consequence of the change of the nucleotide composition across the sequence (Figure 5).

### Application to real data

A real world scenario where alignment-free sequence clustering is desirable is assembly of an HTS run containing a large number of reads. It can be the case that the available computational resources (in particular, memory) are not sufficient to perform a direct assembly. In such instances one may need to split the reads into several sets and assemble each set individually, merging the contigs afterwards. We explore the random splitting and the educated splitting using alignment-free clustering of an Illumina run containing 22 million cDNA reads from a nasal swab. The results are shown in Table 9. It turns out that the educated splitting results in a better assembly (more reads mapping back onto contigs). The difference between the hard EM and the *k*-means partitioning is rather small, and these two partitionings improve assembly compared to the random splitting. The soft EM leads to a better assembly than both hard EM and *k*-means partitioning. The reason for this is that the soft EM algorithm allows a single read to be assigned to multiple clusters simultaneously. This provides more possibilities for the reads from the same contig to be clustered together and consequently assembled. In fact, for a small value of the velvet hash length (namely, 21) the soft EM partitioning results in more reads mapping back to contigs than assembling the run as a whole. We speculate that the explanation for this observation is that the small value of the hash length results in a larger number of contigs at the cost of specificity. Partitioning the reads in an educated way makes assembly of each subset more specific.

**Table 9:** Assembly statistics for clustering and random splitting of a real sample

**Splitting**	**Mapped reads**	**Total bp in contigs**	**Number of contigs**	**n50**
Velvet assembly, hash length 21				
All reads	1507427 (6.99%)	470740477	5650689	79
Soft EM, 2 clusters	1555663 (7.21%)	492995773	5928907	52
EM, 2 clusters	1458627 (6.76%)	453165323	5454404	65
*k*-means, 2 clusters	1475586 (6.84%)	455384651	5474129	124
GC content, 2 parts	1455825 (6.75%)	451987554	5437853	70
Random splitting, 2 clusters	1259894 (5.84%)	428174983	5219268	94
Soft EM, 3 clusters	1614090 (7.48%)	528221487	6359119	78
EM, 3 clusters	1429190 (6.63%)	443042444	5343548	55
*k*-means, 3 clusters	1439961 (6.68%)	443713631	5347679	77
GC content, 3 parts	1397108 (6.48%)	436515238	5278594	98
Random splitting, 3 clusters	1036477 (4.81%)	392638398	4878611	48
Velvet assembly, hash length 31				
All reads	2327126 (10.79%)	290798616	2578825	100
Soft EM, 2 clusters	2263596 (10.50%)	292536888	2643061	204
EM, 2 clusters	2112597 (9.79%)	266185624	2412045	126
*k*-means, 2 clusters	2129306 (9.87%)	267875650	2424380	86
GC content, 2 parts	2106489 (9.77%)	265677735	2407873	100
Random splitting, 2 clusters	1629402 (7.55%)	222061071	2101527	104
Soft EM, 3 clusters	2269196 (10.52%)	310107203	2839376	226
EM, 3 clusters	2002261 (9.28%)	255782318	2354233	86
*k*-means, 3 clusters	2006030 (9.30%)	256111968	2358231	114
GC content, 3 parts	1934436 (8.97%)	247356556	2283296	106
Random splitting, 3 clusters	1257062 (5.83%)	184143812	1807765	141
Velvet assembly, hash length 41				
All reads	1403308 (6.51%)	118746013	848180	127
Soft EM, 2 clusters	1289123 (5.98%)	110992860	805140	188
EM, 2 clusters	1182223 (5.48%)	99860264	725769	129
*k*-means, 2 clusters	1191102 (5.52%)	100436034	728680	125
GC content, 2 parts	1182618 (5.48%)	100638247	733416	127
Random splitting, 2 clusters	839681 (3.89%)	73260257	558661	83
Soft EM, 3 clusters	1275142 (5.91%)	114111918	836929	156
EM, 3 clusters	1081154 (5.01%)	92510990	683516	169
*k*-means, 3 clusters	1078651 (5.00%)	92021168	679148	136
GC content, 3 parts	1027385 (4.76%)	86928363	641027	136
Random splitting, 3 clusters	622242 (2.88%)	55079268	435757	149

Statistics of assembly of real sequencing data. 21,568,249 reads from an Illumina run on a nasal swab cDNA were assembled with and without splitting the sample. Splitting into 2 or 3 clusters was performed randomly as well as using the soft and hard clustering techniques studied in the present work.

### afcluster software

We implemented *afcluster* software for centroid based alignment-free clustering of nucleotide sequences based on word (*n*-mer) counts. Word counts can be computed using overlapping or non-overlapping *n*-mers, optionally concatenating the sequence together with its reverse complement. Where no reading frame is found we recommend using overlapping *n*-mer counts and/or stacking with the reverse complement. Non-overlapping *n*-mer counts can be used to compare the codon usage of coding sequences.

Implemented clustering algorithms include expectation maximization algorithm, *k*-means algorithm as well as centroid algorithms using different distance types: *L*_2_ distance, *d*_2_ distance, $d_{2}^{*}$ distance, symmetrized KL divergence, *χ*^2^ distance. One can also perform consensus clustering. In this case regular clustering is performed a specified number of times, and the consensus partitioning is built based on patterns of individual samples clustering together. Consensus clustering mitigates the dependence of the resulting partitioning on the random initialization inherent to centroid-based methods. However, this is achieved at the cost of $O(N^{2}\log N)$ time complexity and $O(N^{2})$ space complexity for input consisting of *N* sequences.

The software also allows soft EM clustering, in which case each sequence is only assigned to each cluster with some probability. This method gives some estimate of the clustering accuracy without the overhead of the consensus clustering. The ability to simultaneously assign the same sequence to several clusters is also useful when splitting a sample before performing assembly.

*afcluster* software is implemented in C++. It has been compiled and tested using GNU GCC. The tool is open source under the GNU GPL license. It is publicly available from https://github.com/luscinius/afcluster as a source code together with the documentation. It is also available as Additional file 1.

## Conclusions

Alignment-free sequence clustering is a useful tool for sequence analysis. However, it has the limitations found with other clustering algorithms based on word counts. A major potential confound is assumption that for any given gene or organism there is a consistent frequency distribution for counted words. However, there are examples where word frequencies change across the same genome [7-9]. Also, viral genomes are systematically affected by the interaction with the host which leads to the host mimicry [10,11]. A separate study would be required to address these issues.

Centroid based clustering offers the linear time and space complexity, which is critical for large datasets; in particular, HTS reads. Even though the hard expectation maximization algorithm using the Kullback-Leibler divergence as a log likelihood function shows superior performance in some cases, it is computationally feasible to use the *k*-means algorithm as the time gain outweighs the possible accuracy loss. It also turns out that it is sufficient to use short word sizes (*n* = 1 or *n* = 2). Soft expectation maximization clustering is more effective than the hard expectation maximization as it allows to assign a read to more than one cluster simultaneously. Application to a real dataset shows that the soft EM algorithm is especially effective in the context of the HTS read assembly.

## Methods

### Kullback–Leibler divergence is the log likelihood for the word counts

Kullback-Leibler (KL) divergence has a natural probability theory interpretation as a limiting case of the multinomial distribution. In particular, it was used in the context of alignment-free sequence comparison in the work [12];

Under this model one assumes that the counted *n*-mers are drawn from a pool **q** with frequencies of each *n*-mer being *q*_*i*_, index *i* numbering all possible *n*-mers and running from 1 to 4^>*n*^. In other words, the model assumes that the words in a sequence are independent, and the probability of appearance of a particular word at a given position is position independent. The probability of appearance of the word *i* at a given position is *q*_*i*_; *i* = 1,…,4^*n*^. Under these assumptions the probability of obtaining *n*-mer count vector **c** is given by the multinomial distribution^2^:

(1) $$P(c|q)=\frac{L!}{c_{1}!\ldots c_{4^{n}}!}\overset{4^{n}}{\underset{i=1}{\prod }}q_{i}^{c_{i}}.$$

We denote $\sum_{i}c_{i}=L$ — the total number of words in a sequence. For sufficiently large counts one can use the Stirling’s approximation,

$$c!\approx \sqrt{2\pi c}{\left(\frac{c}{e}\right)}^{c},\;c\gg 1;$$

which yields

(2) $$P(c|q)\approx \sqrt{2\pi n}\overset{4^{n}}{\underset{i=1}{\prod }}\frac{1}{\sqrt{2\pi c_{i}}}{\left(\frac{Lq_{i}}{c_{i}}\right)}^{c_{i}}.$$

Denote the normalized counts as

$$p_{i}=\frac{c_{i}}{L};$$

consequently, the log of the probability is

(3) $$\log P(p|q)\approx L\overset{4^{n}}{\underset{i=1}{\sum }}p_{i}\log\frac{q_{i}}{p_{i}}=-LD_{\text{KL}}(p|q).$$

The KL divergence between the frequency distributions **p** and **q** is:

(4) $$D_{\text{KL}}(p|q)=\overset{4^{n}}{\underset{i=1}{\sum }}p_{i}\log\frac{p_{i}}{q_{i}}.$$

When the difference between *p*_*i*_ and *q*_*i*_ is small, this probability distribution reduces to the multivariate normal distribution^3^,

(5) $$\begin{array}{ll} \;\log P(c|q) & =L\overset{4^{n}}{\underset{i=1}{\sum }}p_{i}\log\left(1+\frac{q_{i}-p_{i}}{p_{i}}\right)\\ & \approx L\overset{4^{n}}{\underset{i=1}{\sum }}p_{i}\left(\frac{q_{i}-p_{i}}{p_{i}}-\frac{1}{2}\frac{{\left(q_{i}-p_{i}\right)}^{2}}{p_{i}^{2}}\right)\\ & =-\overset{4^{n}}{\underset{i=1}{\sum }}\frac{{\left(c_{i}-Lq_{i}\right)}^{2}}{2Lq_{i}}. \end{array}$$

We have used the Taylor expansion for the natural logarithm:

(6) $$\log(1+x)=x-\frac{1}{2}x^{2}+O(x^{3}),$$

dropping the cubic and higher terms.

Interpretation of the formula (3) in the context of clustering is as follows. When we have several candidate pools **q**^*α*^ (“centroids”), KL divergence *D*_KL_(**p**|**q**^*α*^) gives the log odds ratio for a sequence having the vector of counts **c** to be attributed to centroid *α*. Therefore the ML estimate of a centroid is obtained by minimizing the KL divergence. We employ this relation within the framework of expectation maximization clustering.

### Expectation maximization clustering

The problem of clustering is the challenge of partitioning a dataset of *N* points into *K* subsets (clusters) such that the similarity between the points within each subset is maximized, and the similarity between different subsets is minimized. The measure of similarity can vary depending on the data. Generally the clustering problem is computationally hard. However, there exist heuristic clustering algorithms that run in polynomial time. Most common clustering approaches are hierarchical clustering, *k*-means type (centroid based) clustering [13] and density based clustering [14]. Each of these approaches possesses its own advantages and disadvantages.

Hierarchical clustering does not require one to specify the number of clusters a priori. Instead it produces the linkage tree, and the structure of the tree (in particular, branch lengths) determines the optimal number of clusters. However, the complexity of hierarchical clustering is at least $O(N^{2})$. Density based algorithms (e. g., DBSCAN [14]) can find clusters of arbitrary shape as well as identify outliers. They do not require the prior specification of the number of clusters. Run time is O(NlogN). Centroid based approaches (*k*-means, expectation maximization) have a linear run time. Prior specification of the number of clusters is required, and results depend on the initialization of the algorithm.

In the present work we focus on centroid based technique. Our rationale for this is as follows. First, there exists a natural likelihood function for the word counts, which allows one to perform EM clustering. Also, the space of word counts possesses a natural notion of a centroid: for a set of sequences which belong to the same cluster one adds all the words within them; and the resulting frequencies yield the cluster centroid. Second, linear run time is critical for large datasets (in particular, HTS data).

EM is a generalization of *k*-means algorithm. The number of clusters *K* needs to be specified in advance. For the execution of the algorithm on *N* sequences one needs the following variables: centroids **q**^*α*^, *α* = 1,…,*K*; and assignments (“latent data”) *z*^*a*^, *a* = 1,…,*N*. The algorithm consists of the two steps repeated iteratively until it converges. 

1. Expectation step: given the current centroids **q**^*α*^, compute the new values of *z*^*a*^ so that the log likelihood L is maximized.

2. Maximization step: given the current assignments *z*^*a*^, compute the new values of **q**^*α*^ so that the log likelihood L is maximized.

This procedure guarantees that the log likelihood is non-decreasing at each step. Note that Equation (3) implies that the log likelihood is bounded from above by zero. These two facts imply that the algorithm converges^4^. In terms of the the variables **q**^*α*^ and *z*^*a*^ the log likelihood is

(7) $$L=-\overset{N}{\underset{a=1}{\sum }}L_{a}D_{\text{KL}}(p^{a}|q^{z^{a}})\equiv -\overset{N}{\underset{a=1}{\sum }}L_{a}\overset{4^{n}}{\underset{i=1}{\sum }}p_{i}^{a}\log\frac{p_{i}^{a}}{q_{i}^{z^{a}}}.$$

We denote the total number of words in the *a*’th sequence as *L*_*a*_. Consequently, expectation step reassigns each point to its closest centroid:

(8) $$z^{a}=\underset{\alpha }{\text{arg min}}D_{\text{KL}}(p^{a}|q^{\alpha }).$$

Centroids are updated during the maximization step as follows:

(9) $$q_{i}^{\alpha }=\frac{\sum_{a=1}^{N}\delta_{z^{a},\alpha }c_{i}^{a}}{\sum_{a=1}^{N}\sum_{j=1}^{4^{n}}\delta_{z^{a},\alpha }c_{j}^{a}}.$$

Here we have introduced the Kronecker delta symbol:

(10) $$\delta_{\alpha \beta }=\left\{\begin{array}{ll} 1, & \alpha =\beta \\ 0, & \alpha \neq \beta \end{array}\right)$$

This prescription exactly corresponds to the natural notion of a centroid: one adds all the words counts within a cluster to obtain the total count vector and normalizes this vector. Detailed derivation of Equation (9) is presented in Appendix 1.

The EM algorithm depends on initialization. In other words, depending on the initial centroid assignment the algorithm may converge to a partitioning that is only locally optimal. One of the ways to minimize the impact of the random initialization is to perform clustering several times using different initializations. This results in several partitionings, and then the one which maximizes the likelihood function is chosen. In the framework of *k*-means clustering selecting the partitioning with the minimal distortion leads to such maximization. Distortion is the sum of the intra-cluster variances for all the clusters. Using KL divergence as a likelihood function, one arrives at the modified definition of distortion:

(11) $$D=\underset{a}{\sum }L^{a}D_{\text{KL}}(p^{a}|q^{z^{a}}).$$

Note that in the limit when the likelihood function reduces to the Gaussian one, our EM algorithm reduces to Gaussian mixture EM. In this case in the light of the formula (5) our definition of distortion reduces to the regular one.

### Alternative distance (pseudo-likelihood) functions

We also explore some other distance functions, such as *d*_2_ and $d_{2}^{*}$[6,15,16]. We are not aware of their direct probabilistic interpretation as a likelihood function. Nevertheless, they represent some distances; i. e., they can serve as some measure of a degree of dissimilarity between the two sequences. One can operate in terms of a distortion function as a measure of the quality of the partitioning. In the case of the EM clustering of *k*-means clustering, distortion equals the negative log likelihood. If one can prove that the analogs of both expectation and maximization steps lead to a decrease of distortion, this provides the basis for the convergence of the clustering algorithm.

#### ***d***_**2**_** distance**

*d*_2_ distance between the two vectors is defined as 1− cos *θ*, where *θ* is the angle between these vectors:

(12) $$d_{2}(c,q)=1-\frac{c\cdot q}{∥c∥∥q∥}.$$

Here ∥**v**∥ denotes the norm of the vector **v**:

(13) $$∥v∥=\sqrt{\overset{4^{n}}{\underset{i=1}{\sum }}v_{i}^{2}},$$

and the dot denotes the dot product:

(14) $$c\cdot q=\overset{4^{n}}{\underset{i=1}{\sum }}c_{i}q_{i}.$$

One can define the distortion function as

(15) $$D=\overset{N}{\underset{a=1}{\sum }}d_{2}(c^{a},q^{z^{a}})=N-\overset{N}{\underset{a=1}{\sum }}\frac{c^{a}\cdot q^{z^{a}}}{∥c^{a}∥∥q^{z^{a}}∥}.$$

In the context of *d*_2_ distance it is natural to normalize the word counts for centroids and individual sequences so that they have a unit norm: ∥**p**∥ = ∥**q**∥ = 1.

EM algorithm can be generalized to use the *d*_2_ distance as follows. During the expectation step one assigns each sequence to the closest (in terms of the *d*_2_ distance) centroid. During the maximization step one updates the centroids as follows:

(16) $$q^{\alpha }=\frac{\sum_{a=1}^{N}\delta_{z^{a}\alpha }p^{a}}{∥\sum_{a=1}^{N}\delta_{z^{a}\alpha }p^{a}∥}.$$

We assume that the word counts for individual sequences are normalized so that ∥**p**^*a*^∥ = 1. Equation (16) is derived in Appendix 1. This procedure ensures that at each step the distortion is non-increasing. The distortion is bounded by zero from below. These two facts ensure the convergence of the algorithm. Equations (12) and (16) imply that the value of the *d*_2_ distance and the updated positions of the the centroids only depend on the normalized word counts. Consequently, the algorithm makes no distinction between the short and the long sequences.

#### $d_{2}^{*}$ distance

$D_{2}^{*}$ distance was introduced in works [15], [16]. Its modification with suitable normalization for comparing short sequences was introduced in work [6] and called $d_{2}^{*}$. This distance computation of expected word frequencies using the zero order Markov model and standardization of the observed word counts. In the context of centroid based clustering it can be formulated as follows.

1. For a given cluster count the frequencies of single nucleotides (1-mers) within the union of all sequences within the cluster.

2. Compute the vector of expected frequencies of *n*-mers **Q** using zero order Markov model. Under this prescription the expected frequency of *n*-mer is the product of the frequencies of individual characters.

3. For a vector of raw counts **x** define the corresponding standardized vector $\tilde{x}$ as

(17) $$\tilde{x_{i}}=\frac{x_{i}-Q_{i}\sum_{j=1}^{4^{n}}x_{j}}{\sqrt{Q_{i}\sum_{j=1}^{4^{n}}x_{j}}}.$$

4. Denote the word count vector of all sequences within a cluster as **x**; then the distance between the centroid of this cluster and a sequence with the word count vector **c** is

(18) $$d_{2}^{*}(c,x)=\frac{1}{2}\left(1-\frac{\tilde{c}\cdot \tilde{x}}{∥\tilde{c}∥∥\tilde{x}∥}\right).$$

Update of sequences’ assignment to clusters is the analog of the maximization step. Update of the expected frequencies is the analog of the expectation step. A priori it is not obvious how to define the distortion so that both expectation and minimization steps lead to a guaranteed decrease in distortion. We leave this question as well as the proof of convergence beyond the scope of the current work.

#### ***χ***^***2***^** distance**

Standardization procedure as defined in Equation (17) is inspired by the Poisson distribution where mean equals variance. Following a similar logic, we introduce the *χ*^**2**^ distance:

(19) $$\chi^{2}(c,Q)=\overset{4^{n}}{\underset{i=1}{\sum }}\frac{{\left(c_{i}-Q_{i}L\right)}^{2}}{Q_{i}L},\;L=\overset{4^{n}}{\underset{i=1}{\sum }}c_{i}.$$

Despite the apparent similarity of this definition with Equation (5), the frequency vector **Q** is the expected vector computed from the zero order Markov model (the same way as it was computed in the calculation of $d_{2}^{*}$ distance).

#### Symmetrized Kullback-Leibler divergence

This distance is the symmetrized Kullback-Leibler divergence:

(20) $$D_{\text{KL}}^{S}(p|q)=\overset{4^{n}}{\underset{i=1}{\sum }}(p_{i}-q_{i})\log\frac{p_{i}}{q_{i}}.$$

It assumes that **p** and **q** are normalized frequency vectors:

(21) $$\overset{4^{n}}{\underset{i=1}{\sum }}p_{i}=\overset{4^{n}}{\underset{i=1}{\sum }}q_{i}=1.$$

### Consensus clustering

Centroid based and hierarchical clustering techniques can be combined in consensus clustering. In this approach centroid based clustering is performed a number of times, each time randomly selecting a fraction of samples into the bootstrap dataset. After that the distance matrix is formed as

(22) $$\begin{array}{l} \;D_{\text{ij}}=1\\ -\frac{\#(\text{times}\;i\;\text{and}\;j\;\text{were}\;\text{clustered}\;\text{together})}{\#(\text{times}\;i\;\text{and}\;j\;\text{were}\;\text{in}\;\text{the}\;\text{same}\;\text{dataset})}. \end{array}$$

Hierarchical clustering is performed with distance matrix *D*_*ij*_. This approach is computationally expensive as complexity of the distance matrix construction is $O(N^{2})$, and the complexity of the hierarchical clustering using average linkage is $O(N^{2}\log N)$ for an input of *N* sequences.

### Recall rate

Consider a set of HTS reads originating from several genes (contigs). Grouping together reads originating from the same gene provides a natural partitioning of the read set. Recall rate is a measure of how well the clustering agrees with this natural partitioning. In other words, the recall rate provides a measure of how well the reads from the same contig cluster together. It is defined as follows. Consider reads originating from some gene *G*. For example, if the number of clusters is *K* = 4 and 40% of reads from *G* are assigned to cluster 1, 20% of reads from *G* are assigned to cluster 2, 10% of reads from *G* are assigned to cluster 3, 30% of reads from *G* are assigned to cluster 4; the recall rate is *R*_*G*_ = 40%.

Generally, assume that there are *K* clusters, and consider reads originating from some gene *G*. Denote *f*_*k*_ the fraction of all reads originating from *G* which are assigned to the cluster *k*. Recall rate for gene *G* is

(23) $$R_{G}=\max(f_{1},\ldots ,f_{k}).$$

Recall rate provides a measure of how clustering interferes with assembly. In particular, when the recall rate is *R*_*G*_ = 1, all reads from gene *G* get assigned to the same cluster; and the contig for *G* can be assembled from just one cluster with no losses.

We performed a numerical experiment to estimate the dependence of the recall rate on the read length and the clustering method. We generated 50 sets of human RNA sequences, each set containing 1000 sequences randomly chosen from the set of the reference sequences. We required that the length of each sequence is at least 500 bp and at most 10000bp. After that we simulated reads of length 30, 50, 75, 100, 150, 200, 250, 300, 400bp from each of these 50 sets using Mason [17]. Each read set contained 100000 reads. Mason options used were illumina -N 100000 -n READLENGTH -pi 0 -pd 0 -pmm 0 -pmmb 0 -pmme 0. This way we obtained a total of 450 simulated read sets: one set for each of the 50 gene sets and 9 values of the read length. To study the dependence of the recall rate on the sequencing error rate for each of the 50 gene sets we generated 100000 reads of length 200 and error rate 0.001, 0.005, 0.01, 0.02, 0.03, 0.04, 0.05. Mason options used were illumina -N 100000 n 200 -pi 0 -pd 0 -pmm ERRORRATE -pmmb ERRORRATE -pmme ERRORRATE. This way we obtained 350 simulated read sets: one set for each of the 50 gene sets and 7 values of the error rate. To study the dependence of the recall rate on the depth of coverage (total number of reads) we simulated read sets with 200000, 150000, 100000, 75000, 50000, 30000, 20000, 10000, 5000 reads. Mason options used were mason illumina -N NUMREADS -n 200 -pi 0 -pd 0 -pmm 0 -pmmb 0 -pmme 0. This way we obtained 450 simulated read sets: one read set for each of the 50 gene sets and 9 values of the number of reads.

We performed hard EM clustering, *k*-means clustering, *L*_2_ clustering and *d*_2_ clustering and computed the recall rate for each gene in each read set. The results show that the EM algorithm exhibits a higher recall rate than that of *k*-means algorithm. For *k*-means clustering we used the implementation available in *scipy*[18] package.

### Soft EM clustering

For the execution of the algorithm one needs the following variables: centroids **q**^*α*^ and probabilities $Z_{a}^{\alpha }$ for observation point *a* to be associated with cluster *α*. EM algorithm iteratively updates probabilities $Z_{a}^{\alpha }$ starting from centroid locations, and then updates centroid locations **q**^*α*^ using the updated probabilities $Z_{a}^{\alpha }$. These steps are performed as follows.

Given a set of centroids **q**^*α*^ and observations (count vectors) **c**^*a*^, the probability for observation *a* to be associated with centroid *α* is

(24) $$Z_{a}^{\alpha }=\frac{P(p^{a}|q^{\alpha })}{\sum_{\beta }P(p^{a}|q^{\beta })},$$

as it follows from Bayes’ theorem. In the “soft” EM algorithm $Z_{a}^{\alpha }$ can take fractional values, calculated according to Equation (24)^5^.

Given the probabilities $Z_{a}^{\alpha }$, one updates centroid locations by maximizing the log likelihood expectation

(25) L(q)=E[logP(p|q)].

When written explicitly it becomes

(26) $$L=-\overset{K}{\underset{\alpha =1}{\sum }}\overset{N}{\underset{a=1}{\sum }}Z_{a}^{\alpha }L_{a}D_{\text{KL}}(p^{a}|q^{\alpha }).$$

Here we denote the number of clusters by *K* and the number of sequences by *N*. In our conventions Greek index *α* runs over the different clusters, Latin index *a* runs over different sequences, and Latin index *i* runs over different *n*-mers. As derived in Appendix 1, centroids are computed as follows:

(27) $$q_{i}^{\alpha }=\frac{1}{\Lambda_{\alpha }}\overset{N}{\underset{a=1}{\sum }}Z_{a}^{\alpha }c_{i}^{a},$$

where

(28) $$\Lambda_{\alpha }=\overset{4^{n}}{\underset{i=1}{\sum }}\overset{N}{\underset{a=1}{\sum }}Z_{a}^{\alpha }c_{i}^{a}.$$

Note that Equation (27) conforms to the intuitive notion of centroid in terms of the word counts. Namely, word counts from all the sequences in the cluster are added up (with some weights in soft EM), and the resulting frequencies are calculated.

As explained, soft EM algorithm assigns the read *a* to the cluster *α* with some probability $Z_{a}^{\alpha }$. Choice of a confidence threshold *ε* produces a set of clusters: read *a* is a member of cluster *α* if $Z_{a}^{\alpha }\geq \varepsilon$. Note that the clusters are possibly overlapping; i. e., one read can be assigned to multiple clusters simultaneously.

### ROC curve for soft EM clustering

Consider a group of reads coming from the same origin (e. g., the same gene or the same organism). A perfect alignment-free classification would assign them to a single cluster (possibly containing some other reads). Let us assume that we know the origin of each read. A choice of some fixed value for the cutoff *ε* will assign each read to zero or more clusters. We consider the cluster which embraces the largest part of the reads from gene *G* to be the “correct” assignment for the reads originating from this gene. For example, assume that we have *K* = 4 (overlapping) clusters, containing 40%, 35%, 35% and 10% of the reads correspondingly. Then the first cluster is the “correct” assignment that would be attributed to all the reads from gene *G* if the clustering algorithm were perfect.

The true positive rate (recall rate) is

(29) $$\text{TPR}=\frac{\#(\text{reads}\;\text{correctly}\;\text{assigned})}{\#(\text{reads})}.$$

We define the false positive rate as

(30) $$\text{FPR}=\frac{\#(\text{reads}\;\text{incorrectly}\;\text{assigned})}{\#(\text{reads})}.$$

A read is considered “incorrectly” assigned if it is assigned to at least one cluster different from the correct one. Note that for some values of the threshold *ε* the same read can be simultaneously assigned to a correct and an incorrect cluster, thus producing both a true and a false positive. In the limit *ε* → 0 each read is assigned to each cluster (FPR=TPR=1). In the limit *ε* → 1 neither read gets assigned to any cluster (FPR=TPR=0).

Dependence of TPR vs FPR as *ε* changes from 0 to 1 gives an analog of the ROC curve^6^. Performance of the algorithm is characterized by the area under the curve (AUC).

### Assembly of real data

Reads from an Illumina run on cDNA of a nasal swab were taken. After filtering out the low quality and the low complexity reads 21,568,249 100bp single end reads were left. Velvet [19] assembly was performed with the default settings. Velveth command line was velveth Assem HASHLENGTH -short -fasta INPUTFILE. Velvetg command line was velvetg Assem. Values of the has length were 21, 31, 41. Assembly was performed on the complete read set as well as on subsets obtained as a result of alignment-free clustering of the reads. Hard clustering was performed 5 times, and the partitioning with the minimal distortion was chosen. Soft clustering was performed once. Confidence cutoff for the soft clustering is *ε* = 0.05. For every splitting of the read set all the contings generated from individual parts were merged together. After that the original reads were mapped back onto the contings using bwa[20] and allowing up to 2 mismatches. The number of reads which map back onto the contigs is a measure of the quality of assembly. It takes care of the possible duplicate contigs which may be formed when assembling separate parts of the sample.

### Sequence data

Reference sequences for human mRNA genes were obtained from NCBI RefSeq ftp site, http://ftp.ncbi.nih.gov/refseq/H_sapiens/mRNA_Prot/. Data were downloaded from NCBI on Apr 09 2013. Sequences for the bacterial recA, dnaA, rpsA and 16S rRNA genes used in the simulation were extracted from *streptococcus pneumoniae* genome, [GenBank:NC_003028]. Viral sequences used in the simulation are [GenBank:NC_001477, NC_001943, NC_000883, NC_015783, NC_001806, NC_003977, NC_001802]. We concatenate all the segments of segmented viruses (rotavirus [GenBank:NC_011500, NC_011506, NC_011507, NC_011508, NC_011510, NC_011501, NC_011502, NC_011503, NC_011504, NC_011509, NC_011505], Lujo virus [GenBank:FJ952385, FJ952384] and influenza virus). For influenza virus we use the sequence of the vaccine strain, A/California/7/2009.

## Endnotes

^1^ Having in mind application of the clustering methods to high throughput sequencing data, we use the words “read” and “sequence” interchangeably throughout the paper.

^2^ Our notations are as follows. We use boldface letters to denote the vectors in the 4^*n*^-dimensional space of the word counts (e. g., **p**, **q**), and we use regular letters to denote the individual components of such vectors (e. g., *p*_*i*_, *q*_*i*_). We denote the vector of raw word counts by **c**, its components are integers. We denote the coordinates of a centroid by **q**, and the normalized word counts by **p**. Normalization of **p** and **q** means that

$$\overset{4^{n}}{\underset{i=1}{\sum }}p_{i}=\overset{4^{n}}{\underset{i=1}{\sum }}q_{i}=1$$

unless otherwise specified. Also, *P*(**c**|**q**) and *P*(**p**|**q**) denote the likelihood of obtaining raw counts **c** or normalized counts **p** under our model if the sequence is assigned to the centroid with coordinates **q**. Note that *P*(**c**|**q**) and *P*(**p**|**q**) denote the same quantity. Either notation is used depending on the context.

^3^ Note the constraint $\sum_{i}c_{i}=L$.

^4^ Note that an empty cluster can be formed at one of the steps. In this case the algorithm fails.

^5^ Recall that in the “hard” EM algorithm $Z_{a}^{\alpha }$ can only be 0 or 1 (each point has to be assigned to exactly one cluster). In this case one finds the maximum likelihood estimate *α*(*a*) for each *a* and sets $Z_{a}^{\beta }=\delta_{\beta ,\alpha (a)}$. Note that this might lead to a formation of an empty cluster after one of the iterations.

^6^ This dependence is not a ROC curve in the sense of the standard definition since the clustering does not generally produce a binary classification.

## Appendix 1

### Evaluation of centroids during the maximization step

Prescription for updating centroids **q**^***α***^ during the maximization step can be derived as follows. One needs to minimize the log likelihood as defined in Equation (7) w. r. t. the variables $q_{i}^{\alpha }$ under the constraint $\sum_{i}q_{i}^{\alpha }=1$ for all *α*. The log likelihood function is a sum of log likelihood functions for different clusters. One therefore can independently maximize it w. r. t. each centroid. Without a loss of generality one can assume that the sequences assigned to a given cluster are numbered 1,…,*M*. Maximizing the likelihood can be done with the help of introducing a Lagrange multiplier Λ and maximizing the new function:

(31) $$\tilde{L}=-\overset{M}{\underset{a=1}{\sum }}L_{a}\overset{4^{n}}{\underset{i=1}{\sum }}p_{i}^{a}\log\frac{p_{i}^{a}}{q_{i}}-\Lambda \left(\overset{4^{n}}{\underset{i=1}{\sum }}q_{i}-1\right).$$

We have dropped the superscript *α* since we only consider one cluster and one centroid. Differentiating w. r. t. *q*_*i*_ (*i* = 1,…,4^*n*^) and equating to zero yields a set of equations:

(32) $$\frac{\partial \tilde{L}}{\partial q_{i}}=\frac{\sum_{a=1}^{M}L_{a}p_{i}^{a}}{q_{i}}-\Lambda =0.$$

These equations imply

(33) $$q_{i}=\frac{\sum_{a=1}^{M}L_{a}p_{i}^{a}}{\Lambda }.$$

Normalization of **q** implies that

(34) $$\Lambda =\overset{M}{\underset{a=1}{\sum }}L_{a}p_{i}^{a}.$$

Substituting for Λ yields

(35) $$q_{i}=\frac{\sum_{a=1}^{M}L_{a}p_{i}^{a}}{\sum_{b=1}^{M}\sum_{j=1}^{4^{n}}L_{b}p_{j}^{b}}.$$

Raw word counts $c_{i}^{a}$ are related to the frequencies $p_{i}^{a}$ as $c_{i}^{a}=L_{a}p_{i}^{a}$. This proves Equation (9).

### Evaluation of centroids for ***d***_***2***_** distance**

This derivation follows that for the EM clustering very closely. Distortion as defined by Equation (15) is a sum of distortions of different clusters. We can minimize the distortion in each cluster separately. Assuming that the word count vectors for each sequence have a unit norm, we can write the distortion within a cluster as

(36) $$D=M-\overset{M}{\underset{a=1}{\sum }}p^{a}\cdot q,\;∥q∥=1.$$

Again, without a loss of generality we assume that the sequences within a cluster are numbered from 1 to *M*. We need to minimize D under the constraint that ∥**q**∥^**2**^ = 1. This can be achieved by minimizing the auxiliary function

(37) $$\begin{array}{ll} \;\tilde{D} & =M-\overset{M}{\underset{a=1}{\sum }}p^{a}\cdot q-\Lambda (∥q{∥}^{2}-1)\\ & =M-\overset{M}{\underset{a=1}{\sum }}\overset{4^{n}}{\underset{i=1}{\sum }}p_{i}^{a}q_{i}-\Lambda \left(\overset{4^{n}}{\underset{i=1}{\sum }}q_{i}^{2}-1\right). \end{array}$$

Differentiating $\tilde{D}$ w. r. t. to *q*_*i*_ and equating to zero yields

(38) $$\frac{\partial \tilde{D}}{\partial q_{i}}=-\overset{M}{\underset{a=1}{\sum }}p_{i}^{a}-2\Lambda q_{i}=0.$$

This solves for **q** as^7^

(39) $$q=-\frac{1}{2\Lambda }\overset{M}{\underset{a=1}{\sum }}p^{a},\;\Lambda =-\frac{1}{2}∥\overset{M}{\underset{a=1}{\sum }}p^{a}∥.$$

This proves Equation (16).

### Evaluation of centroids in the soft clustering

We use the same techniques as those used for the hard EM clustering. The difference is that now we cannot consider clusters independently as there is no “hard” assignment of each data point to a single cluster. We add Lagrange multipliers Λ_*α*_ to Equation (26) to account for the constraints $\sum_{i}q_{i}^{\alpha }=1$:

(40) $$\tilde{L}=-\overset{K}{\underset{\alpha =1}{\sum }}\overset{N}{\underset{a=1}{\sum }}Z_{a}^{\alpha }L_{a}\overset{4^{n}}{\underset{i=1}{\sum }}p_{i}^{a}\log\frac{p_{i}^{a}}{q_{i}^{\alpha }}-\overset{K}{\underset{\alpha =1}{\sum }}\left(\Lambda_{\alpha }\overset{4^{n}}{\underset{i=1}{\sum }}q_{i}^{\alpha }-1\right).$$

Differentiating $\tilde{L}$ w. r. t. $q_{i}^{\alpha }$ and equating to zero yields the following set of equations:

(41) $$\frac{\partial \tilde{L}}{\partial q_{i}^{\alpha }}=\frac{1}{q_{i}^{\alpha }}\overset{N}{\underset{a=1}{\sum }}Z_{a}^{\alpha }L_{a}p_{i}^{a}-\Lambda_{\alpha }=0.$$

These equations imply

(42) $$q_{i}^{\alpha }=\frac{1}{\Lambda_{\alpha }}\overset{N}{\underset{a=1}{\sum }}Z_{a}^{\alpha }L_{a}p_{i}^{a}.$$

Imposing normalization constraints on $q_{i}^{\alpha }$ yields

(43) $$\Lambda_{\alpha }=\overset{N}{\underset{a=1}{\sum }}\overset{4^{n}}{\underset{i=1}{\sum }}Z_{a}^{\alpha }L_{a}p_{i}^{a}.$$

This proves Equations (27) and (28).

## Abbreviations

AUC: Area under the curve; bp: base pairs; EM: Expectation maximization; FPR: False positive rate; HTS: High throughput sequencing; KL divergence: Kullback-Leibler divergence; ROC: Receiver operating characteristic; TPR: True positive rate.

## Competing interests

Both authors declare that they have no competing interests.

## Authors’ contributions

AS wrote the algorithms and performed the analyses. Both AS and WIL reviewed the data and wrote the manuscript. Both authors read and approved the final manuscript.

## Supplementary Material

Additional file 1 **Source code and documentation for** ***afcluster* software.** Also available from https://github.com/luscinius/afcluster. Click here for file
